# Using User-Centered Design to Facilitate Adherence to Annual Lung Cancer Screening: Protocol for a Mixed Methods Study for Intervention Development

**DOI:** 10.2196/46657

**Published:** 2023-04-14

**Authors:** Erin A Hirsch, Jamie L Studts

**Affiliations:** 1 Division of Medical Oncology University of Colorado School of Medicine University of Colorado Anschutz Medical Campus Aurora, CO United States; 2 University of Colorado Cancer Center Aurora, CO United States

**Keywords:** health information processing, intervention design, lung cancer, lung cancer screening, LCS, mixed methods, photovoice, user-centered design

## Abstract

**Background:**

Lung cancer is the leading cause of cancer-related death in the United States, with the majority of lung cancer occurrence diagnosed after the disease has already metastasized. Lung cancer screening (LCS) with low-dose computed tomography can diagnose early-stage disease, especially when eligible individuals participate in screening on a yearly basis. Unfortunately, annual adherence has emerged as a challenge for academic and community screening programs, endangering the individual and population health benefits of LCS. Reminder messages have effectively increased adherence rates in breast, colorectal, and cervical cancer screenings but have not been tested with LCS participants who experience unique barriers to screening associated with the stigma of smoking and social determinants of health.

**Objective:**

This research aims to use a theory-informed, multiphase, and mixed methods approach with LCS experts and participants to develop a set of clear and engaging reminder messages to support LCS annual adherence.

**Methods:**

In aim 1, survey data informed by the Cognitive-Social Health Information Processing model will be collected to assess how LCS participants process health information aimed at health protective behavior to develop content for reminder messages and pinpoint options for message targeting and tailoring. Aim 2 focuses on identifying themes for message imagery through a modified photovoice activity that asks participants to identify 3 images that represent LCS and then participate in an interview about the selection, likes, and dislikes of each photo. A pool of candidate messages for multiple delivery platforms will be developed in aim 3, using results from aim 1 for message content and aim 2 for imagery selection. The refinement of message content and imagery combinations will be completed through iterative feedback from LCS experts and participants.

**Results:**

Data collection began in July 2022 and will be completed by May 2023. The final reminder message candidates are expected to be completed by June 2023.

**Conclusions:**

This project proposes a novel approach to facilitate adherence to annual LCS through the development of reminder messages that embrace content and imagery representative of the target population directly in the design process. Developing effective strategies to increase LCS adherence is instrumental in achieving optimal LCS outcomes at individual and population health levels.

**International Registered Report Identifier (IRRID):**

DERR1-10.2196/46657

## Introduction

### Background

Lung cancer is the leading cause of cancer-related deaths in both men and women in the United States [1,2] and is expected to account for approximately 21% of all cancer deaths in 2022 [2]. Most lung cancer occurrences are diagnosed at advanced stages, after the cancer has metastasized, directly contributing to the deadliness of the disease [2]. Lung cancer screening (LCS) with low-dose computed tomography (LDCT) can detect lung cancer in the early stages when curative and less-invasive treatment options exist [3,4]. LCS received a B recommendation from the United States Preventive Services Task Force (USPSTF) in late 2013, prompting private insurance companies to cover the cost of the service for high-risk individuals, defined as individuals aged 55 to 80 years, with a minimum tobacco smoking exposure history of 30 pack-years and currently smoking or have quit within the past 15 years [5]. The Centers for Medicare and Medicaid Services (CMS) subsequently approved reimbursement for LCS in 2015 for Medicare beneficiaries, although they recommended stopping screening at 77 years of age [6]. Recently, the LCS eligibility criteria were expanded by lowering the recommended starting age to 50 years and tobacco exposure to 20 pack-years [7,8]. The USPSTF implemented these recommendations in 2021, followed by Medicare in early 2022.

The efficacy evidence for implementing LCS in routine clinical practice is based on the results of 2 large randomized trials: the National Lung Screening Trial [3] and the Dutch-Belgian Lung Cancer Screening Trial [4]. The results from these trials showed that LCS can reduce lung cancer–specific mortality by at least 20% when performed annually. Importantly, both trials diagnosed more early-stage cancers on follow-up rounds of screening than on the first baseline LDCT scan [3,9], highlighting the importance of sustained engagement with LCS programs. Adherence to multiple rounds of screening exceeded 90% in both clinical trials, further demonstrating the importance of annual screening to maximize the effectiveness of LCS [3,9]. Unfortunately, the translation of yearly LCS participation has emerged as one of the core threats to fully achieving the population health benefits of early lung cancer detection, with real-world annual adherence rates as low as 22% [10]. There is an urgent need for individual- and system-level interventions that focus on increasing the annual adherence rates.

The American College of Chest Physicians recommends that LCS programs develop strategies to maximize compliance with annual screening, including mechanisms to communicate with screening participants [11]. Community and academic LCS programs have reported using reminder letters to address adherence [12-14], and the Community Preventive Services Task Force (established by the United States Department of Health and Human Services in 1996) strongly recommends client reminders for breast cancer screening with mammography and colorectal cancer screening with fecal occult blood test [15]. Research supporting this recommendation found that any type of reminder (live telephone call, automated telephone call, letters, and postcards) increased adherence rates over no reminders, and targeted and tailored materials were superior to generic materials for continued screening participation [16,17].

Although research on established cancer screening modalities serves as a starting point for understanding interventions for reminder preferences, modes, and frequency, the needs and context of the LCS-eligible population must be considered during intervention development. For optimal impact, the unique elements of the LCS and the eligible community must be used to inform efforts to build and sustain engagement with the LCS process. At the center of this milieu is the stigma surrounding cigarette smoking and lung cancer, which is deeply embedded in all levels of society and presents as a barrier for some LCS-eligible candidates to participate in screening [18]. In addition, individuals eligible for LCS often face additional health care disparities, as cigarette smoking is linked to indicators of low socioeconomic status such as rural residence, individuals with Medicaid or being uninsured, and racial and ethnic minority groups [19]. Furthermore, health messages that contain pictures are processed more efficiently [20], highlighting another integral component of LCS communication. Understanding the informational needs and imagery considerations of the LCS-eligible population is crucial for messaging in reminder intervention development.

### Objectives

This paper describes an iterative, mixed methods study that uses the principles of user-centered design and participatory research methods to develop a reminder message intervention specific to annual LCS. This research study will (1) determine how LCS participants process health information regarding screening to identify possible options for reminder message content targeting and tailoring, (2) pinpoint imagery options that engage LCS participants, and (3) use these data to develop adherence to reminder messages for individuals who participate in LCS. Accordingly, the aims of this study are as follows:

- Aim 1: to determine key reminder preferences and demographic and clinical characteristics associated with LCS-specific health processing, informed and facilitated by Cognitive-Social Health Information Processing (C-SHIP) model constructs to guide reminder message content and options for message targeting and tailoring.
- Aim 2: to identify engaging graphic themes drawn from photos selected by LCS participants to facilitate annual adherence aided by meaning and visual appeal.
- Aim 3: to develop and evaluate a pool of candidate reminder messages for multiple platforms (postcard, letter, and SMS text messaging) integrating visual imagery based on LCS experts and participant feedback using the principles of user-centered design.

## Methods

### Design Overview

The goal of this research is to use a theory-informed, multiphase, and mixed methods approach with LCS experts and participants to develop a set of clear and engaging reminder messages to support LCS annual adherence. In a 3-step process, survey data (step 1) and themes for imagery informed by LCS participant photovoice and key informant mixed method interviews (step 2) will inform the development of a pool of candidate reminder messages (step 3) for usability evaluation and testing with LCS experts and participants. This research will be guided by methodological, theoretical, and conceptual frameworks and principles to direct the steps needed to design and evaluate reminder message content, provide guidance to measure relevant constructs about how LCS participants process health information, and inform message context and imagery through engagement with LCS participants.

### Methodologic Framework

**Figure 1:**
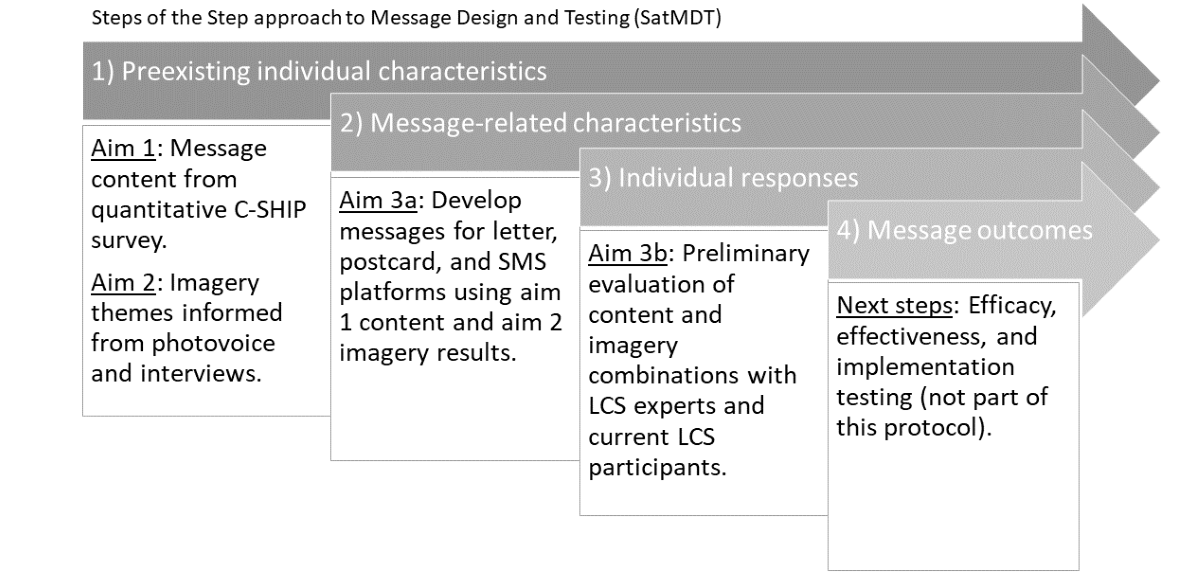
Study approach as guided by the Step approach to Message Design and Testing (SatMDT). C-SHIP: Cognitive-Social Health Information Processing Model; LCS: lung cancer screening.

This research study will be guided by the Step approach to Message Design and Testing (SatMDT), a four-step conceptual process—(1) preexisting individual characteristics, (2) message-related characteristics, (3) individual responses, and (4) message outcomes—that will guide the overall reminder message development, from content identification to outcome evaluation [21]. As shown in Figure 1, step 1 of the SatMDT corresponds to study aims 1 and 2, informing the selection of relevant message content identified from quantitative survey data and further guiding reminder message imagery selection through the photovoice and interview activity. Aim 3 operationalizes SatMDT steps 2 and 3 by first using content results from aim 1 and imagery results from aim 2 to develop 10 to 12 reminder messages with varying visual combinations for each reminder type (ie, letter, postcard, and SMS text messaging). Second, it conducts preliminary testing of the message content and imagery combinations with rapid, iterative feedback with LCS experts and participants. SatMDT step 4 is outside the scope of this study. However, future studies will evaluate the efficacy and effectiveness of reminder messages to improve LCS annual adherence rates in clinical trials and real-world settings and on implementation testing to measure the acceptability and feasibility of using the reminder system in busy clinical settings.

### Theoretical Framework

To find informative and appealing content for the reminder messages that can further be targeted and tailored to the intended recipients, the C-SHIP model [22] will be applied to identify, contextualize, and operationalize the content of the reminder messages for the aim 1 quantitative survey. The C-SHIP model draws from several theoretical frameworks within the fields of health, cognitive, clinical, social, and behavioral psychology to identify health information seeking and processing behaviors, with the main premise detailing that health decision-making processes are the result of an interaction between the individual and the environment [22]. Aim 1 survey data informed by C-SHIP will be collected to assess how LCS participants process health information aimed at health protective behavior to develop content for the reminder messages and pinpoint options for message targeting and tailoring.

### Conceptual Framework and Guidance

Additional research methodology will be guided by user-centered design and participatory photovoice to identify relevant content and imagery through engagement with LCS-eligible individuals and assessing preliminary usability testing. User-centered design is an intervention design approach that encompasses information about the individuals that will be using the intervention in the development and testing processes [23]. The goal of user-centered design is to maximize the usability of the intervention among the population of interest in a real-world context [23]. User-centered design will be used to (1) design the reminder intervention based on the characteristics of LCS participants and (2) refine message content and imagery based on rapid testing and feedback from LCS experts and participants. Consideration of the needs and emotions of LCS participants is vital for effective adherence reminder messaging.

Photovoice is a participatory research method that invites participants to take pictures representing an area of research within their community, allowing areas of concern to be documented and contemplated [24]. Traditional photovoice is completed by providing participants with a camera to capture the visual representation of the concern or research question and is then discussed through critical dialog in focus groups [25]. In this research project, photovoice will be used to invite participants to identify images that represent LCS to them and to identify themes for reminder message imagery through mixed methods interviews with the photovoice participants to understand the selection, likes, and dislikes of each photo.

### Aim 1—Participant Health Information Processing Survey to Inform Message Content

#### Participants

The study participants will be recruited from three established LCS programs across the Colorado Front Range: (1) UCHealth University of Colorado Hospital in Aurora, Colorado; (2) UCHealth South in Colorado Springs, Colorado; and (3) UCHealth North in Fort Collins, Colorado. All LCS participants screened at these sites that meet the CMS or USPSTF eligibility guidelines, are English speaking, and have a screening-specific LDCT scan with results recommending 1-year (annual) follow-up (Lung-RADS 1 or 2) will be invited to complete the aim 1 health information processing survey 1 to 2 weeks after their LDCT. LCS participants who do not agree or consent to complete the survey or whose screening LDCT recommends shorter-term follow-up (Lung-RADS 3 or 4) will be excluded.

#### Procedure

##### Recruitment

All LCS participants that are screened in the 3 recruitment programs and have LDCT results recommending annual screening follow-up will be identified from clinical databases used to manage each program (Powerscribe Lung Cancer Screening developed by Nuance or Lung Cancer Screening Eon Patient Management developed by Eon Health). Additional variables, including Lung-RADS score, sex, race, ethnicity, smoking status, and pack-year information, will be abstracted from the clinical database to describe the study population and to report any differences between respondents and nonrespondents.

##### Survey Distribution

Potential study participants will be emailed a link for a REDCap (Research Electronic Data Capture; Vanderbilt University; electronic data capture hosted at University of Colorado Anschutz Medical Campus) web-based survey if an email is available in the electronic health record or a mailed paper version of the survey if only a postage address is available. The Tailored Design Method [26] will be used to maximize the response rate; specifically, enrolled participants will be contacted on multiple attempts if records indicate that the survey has not been completed, and respondents will receive US $40 compensation on the completion of the survey. Potential participants who received the survey by email will be contacted up to 4 times, 3 times by email and once by postal mail. This strategy has been reported to help increase survey response rates for individuals who do not respond to web-based surveys. Potential study participants who receive the survey by postal mail will be contacted up to 3 times.

Interested participants will be asked to acknowledge the contents of the informational consent form before proceeding with the survey. By completing the survey, respondents will agree to participate in the research study.

##### Sample Size

Using a 2-sample proportion test and assuming a minimum sample size of 125 survey respondents, there will be 80% power to detect a minimum difference of 20% for a *χ*^2^ test with 1 *df* or 28% for a *χ*^2^ test with 2 *df* between C-SHIP constructs (dependent) and survey respondent characteristics (independent) at an α level of .05. Power calculations were performed using PASS 11 (NCSS, LLC) [27]. A minimum of 125 complete surveys will be an achievable goal because the Tailored Design Method [26] will be used to optimize the response rate by offering multiple modes of survey delivery, making multiple contact attempts to nonrespondents, and offering US $40 compensation on the completion of the survey.

#### Measures

Relevant constructs from each C-SHIP domain have been chosen as a foundation for survey questions based on an extensive LCS literature review and identification of how C-SHIP has been applied to breast, cervical, and prostate cancer research [22,28,29]. Multimedia Appendix 1 [30-43] shows each LCS construct relevant to each C-SHIP domain and the scale or measure that will be used to measure each construct. Standardized scales were chosen wherever possible during the development of the aim 1 quantitative survey (LCS Health Beliefs Scale [30], Health Information National Trends Survey [31], Health Perceptions Questionnaire [32], Self-Regulatory Questionnaire for LCS [33], LCS Knowledge Survey [34], Cataldo Lung Cancer Stigma Scale [35], Revised Powe Fatalism Inventory [36], Health Behavior Scale for Cancer Patients [37], Medical Minimizer Maximizer Scale 1 [38], BRIEF Health Literacy Scale [39], Subjective Numeracy Scale [40], 9-item Shared Decision-Making Questionnaire [41], and radiology results from Woolen et al [42]).

The survey instrument contains all close-ended questions and comprises eight sections: (1) health-relevant encodings, (2) health beliefs and expectancies, (3) affects (emotions), (4) health goals and beliefs, (5) self-regulatory competencies, (6) previous screening LDCT and shared decision-making experience (satisfaction and stigma), (7) reminder preferences (timing, modality, and frequency), and (8) demographic and clinical information. The principles of the Tailored Design Method [26] (using simple words, complete sentences, as few words as possible, logical question organization, and easily understood concepts) were used to develop the survey to reduce common survey biases. The survey takes approximately 30 minutes to complete.

#### Analysis

All data from the aim 1 survey will be quantitative, mainly using Likert-type scales. Survey responses that occur more frequently will be prime candidates for reminder message content. Differences in C-SHIP constructs between demographic and clinical characteristics will guide reminder message content, with any statistically significant differences becoming a focus for message targeting and tailoring. To test for differences between C-SHIP constructs and respondent characteristics, the normal distribution of all variables will be evaluated with quantile-quantile plots. Variables with a normal distribution will be treated as continuous, whereas nonnormally distributed data will be categorized or analyzed using nonparametric tests. Univariate differences between the constructs and characteristics will be assessed with a 2-tailed *t* test or ANOVA for continuous variables (or nonparametric tests using ranks) and the chi-square test for categorical variables. The quantitative data collected in aim 1 will directly inform and influence the development of the reminder message content in aim 3.

### Aim 2—Participant Photovoice and Interviews to Inform Themes for Message Imagery

#### Participants

Participants for aim 2 will be recruited from the same LCS programs used for the aim 1 survey. This sampling of participants will be recruited both independently and as a continuation of the aim 1 sample. As the purpose of aim 2 (to inform the imagery that will be selected for reminder messages) is separate from that of aim 1, no bias is expected from involving the same participants for both aims 1 and 2. Participants who completed an LCS LDCT within the past 12 months with results of Lung-RADS 1 or 2 with recommended 1-year (annual) follow-up and are English speaking will be eligible for aim 2. Sampling will be purposive, with emphasis placed on accruing heterogeneous participants (eg, males and females, race and ethnicity, and individuals that currently and formerly smoked cigarettes) for the photovoice activity and subsequent interviews. Participants who do not agree or consent to the photovoice activity and interview or whose screening LDCT recommends shorter-term follow-up (Lung-RADS 3 or 4) will be excluded from aim 2.

#### Procedure

##### Recruitment

The recruitment strategies will be similar to those of aim 1. Participants eligible for aim 2 will have completed an LCS LDCT that recommends 1-year (annual) follow-up within the past 12 months. Potential participants who represent a wide range of demographics and demographic characteristics (purposive sampling) will be sent an informational letter about aim 2 with directions to return the response card in the postage paid addressed envelope if only a postal address is available in the electronic health record or respond to the email to receive more information about participation.

The research team will follow-up with interested individuals using the contact information provided on the response card or email with additional information about participation in aim 2. Owing to the ongoing COVID-19 pandemic, enrollment will be limited to individuals who have access to computers with internet and video and audio capabilities. Participants will be asked to agree to an information sheet and consent electronically in REDCap to participate in aim 2.

##### Participant Photovoice Procedures

After obtaining informed consent, aim 2 participants will be asked to find 3 photos that represent LCS to them and to upload them into a REDCap database to later be used for the interview portion. This approach will use a modified photovoice component for participants to visually capture their idea of LCS by taking photos or finding photos using Google Images. Photovoice is a visual research methodology that traditionally supplies participants with a camera to take a picture that captures the essence of a cultural phenomenon or problem [24]. Photovoice is meant to facilitate community engagement in research and invoke a *voice* for self-perception in a research problem [24]. Using photovoice for LCS adherence will help pinpoint visual imagery that is attractive to a population traditionally stigmatized at individual and societal levels. The photovoice activity instructions will provide prompts about participating in LCS to help them think thoughtfully when choosing photos (Multimedia Appendix 1 [30-43]).

##### Interview Guide, Procedures, and Measures

Photovoice interviews will be conducted via Zoom and will be guided by a semistructured interview guide and use both open- and close-ended questions. Close-ended questions will yield quantitative data, and the open-ended questions will provide qualitative data. Participants will be shown the images they chose for the photovoice component one at a time on Zoom and asked questions about why they chose the image, what the image means to them, and likes and dislikes about the image. The interview questions were modified from the SHOWeD technique, which is commonly used in photovoice studies to help participants discuss the photos they captured [43] (Multimedia Appendix 1 [30-43]). Interview participants will also be asked to rate the image on a scale of 0 to 10, where 0 is “I don’t like this photo” and 10 is “I really like this photo.” At the end of the interviews, participants will be shown images commonly used in current LCS communications (cigarettes, lungs, and smoke) and will be asked about their thoughts, likes, dislikes, and ratings of each image on a scale of 0 to 10 (Multimedia Appendix 1 [30-43]). Probing questions will be used when appropriate. Interviews will be audio recorded and transcribed verbatim. Interview participants will receive US $60 as compensation on the completion of the interview.

##### Sample Size

The participants photovoice aim involves conducting approximately 20 debriefing interviews to reach saturation for photo visual appeal and thematic content. Interviews will be stopped early if saturation is reached before 20 interviews or will continue beyond 20 if the research team concurs that saturation has not been reached.

#### Analysis

Aim 2 interviews will use qualitative and quantitative data collected in parallel from the same interview guide, as described earlier. Following a parallel convergent mixed methods design [44], quantitative and qualitative data will be analyzed separately and then merged to make final conclusions about themes for reminder message imagery. Quantitative data from the closed-ended rating questions will be recorded in a matrix in Microsoft Excel (Microsoft Corporation), and similar responses will be categorized and ranked in order of frequency. Qualitative data from open-ended questions will be analyzed using thematic analysis [45]. Coding will be completed by 2 coders for the first 5 interviews to establish intrareader reliability, calculated using Pearson correlation or κ statistics. Coding will be inductive, meaning that the research team will use codes that emerge from the data. Standardized codes from the first 5 interviews will also be used to inform the development of a codebook that will be used to code the remaining interviews. The study team will meet weekly during the coding process to review and categorize themes and to determine when saturation has been reached. Finally, the team will integrate the results from the qualitative and quantitative data to make rich, multiangled inferences for the determination of the final imagery themes. The integration of the 2 data types will be done using a matrix analysis, in which the quantitative imagery ratings are compared with the qualitative themes. It is expected that the data types will corroborate, with higher-rated imagery containing positive and encouraging themes and lower-rated imagery associated with themes about lower-rated pictures. The matrix design will also offer the opportunity for qualitative data to explain the quantitative ratings and highlight the specific aspects the participants did or did not like regarding high- or low-rated imagery.

### Aim 3—Development and Preliminary Evaluation of Reminder Messages

The purpose of aim 3 is 2-fold: first, to develop a group of reminder message candidates for 3 platforms (ie, letter, postcard, and SMS text messaging) with differing content and imagery combinations, and second, to conduct preliminary usability testing of the messages with LCS experts and participants. Evaluative testing will consist of rapid, iterative feedback cycles, with the refinement of messages occurring after each cycle of 3 experts or participants.

#### Development of Reminder Message Candidates

Reminder messages and content will be developed by the principal investigator (EAH), with final message candidates reviewed and finalized by the full research team. Messages will likely focus on conveying the benefit (“gain-framed”) of annual LCS adherence [46]. Gain-framed messages have been shown to lead to greater knowledge and message recall [47]. Options for reminder message visual imagery based on themes from aim 2 will be chosen to accompany the message text. Messages that include a combination of verbal and visual cues are processed more efficiently than those with text alone [20]. Message content will be guided by messages that have led to increased adherence to repeat mammography [48,49] and messages developed for LCS awareness campaigns [50]. Overall, 10 to 12 reminder messages with differing visual appeals for each reminder type (postcards, letters, and SMS text messages) will be developed to achieve engagement for LCS-eligible individuals. Differences between health information processing constructs by demographic or clinical characteristics found to be statistically significant from aim 1 will be the focus of message targeting and tailoring.

#### Preliminary Testing by LCS Experts

Expert review of the reminder message candidate combinations (content and imagery) will be completed by LCS experts to gauge the message content from a clinical and implementation perspective.

### LCS Expert Study Participants

Experts will be prespecified by the research team before the survey invitation and will include pulmonologists, radiologists, primary care clinicians, advanced practice providers, medical oncologists, and implementation scientists with expertise in LCS. Experts who agree to complete the evaluative survey will be included in aim 3. LCS experts who do not agree to participate in the rating process will be excluded.

LCS experts will be selected a priori based on the knowledge and contacts of the research team. Experts will be contacted via email to introduce the study and ask about their willingness to participate. Every effort will be made to have a balanced panel of experts (ie, pulmonologists, radiologists, advanced practice coordinators, primary care providers, medical oncologists, and implementation specialists). Informed consent will be obtained electronically using REDCap.

### LCS Expert Survey Distribution and Follow-up Procedure

Expert surveys will be closed-ended and administered on the REDCap electronic platform. Respondents will be presented with a mock-up of each message-type and a brief clinical vignette with targeting and tailoring characteristics (if applicable) and asked to rate each message on 6 categories adapted from the Centers for Disease Control and Prevention (CDC) Health Communication Playbook [51] and the National Institutes of Health (NIH) Health Literacy Online [52] guidelines. Message ratings will use a 7-point Likert scale, where 1 is “very poor” and 7 is “very good,” to rate the following categories specific to LCS annual adherence: (1) relevance of the message content, (2) readability of the message content, (3) accuracy of the message content, (4) intent of the message content (conveying screening benefit), (5) memorability of the visual appeal, and (6) overall rating of the message content and visual imagery combination (Multimedia Appendix 1 [30-43]). Experts will be asked to evaluate each message candidate, 10 to 12 for each message-type platform (letter, postcard, and SMS text messaging), or 30 to 36 messages in total.

### LCS Expert Sample Size

Rapid, iterative feedback and refinement of messages will be completed in cycles comprised of 3 experts. It is expected that usability testing will consist of 3 to 4 iterative cycles, with a total of 9 to 12 LCS experts.

### LCS Expert Analysis Plan and Message Refinement

Data from the expert survey will be quantitative. Responses will be entered into a matrix in Microsoft Excel, and similar responses will be categorized and ranked in the order of frequency. Messages with lower ratings will be revised accordingly. Message candidates will be refined based on the feedback of each feedback cycle.

#### Preliminary Evaluation by LCS-Eligible Participants

In addition to expert review, further evaluative testing of the reminder messages will be performed with LCS participants who are close to their annual screening to determine the usefulness, desirability, and value of the reminder message content and imagery combinations.

### LCS-Eligible Study Participants

Message evaluation participants will be recruited solely from the UCHealth University of Colorado Hospital LCS program, with emphasis on participants who are close to their annual screening LDCT (Lung-RADS result of 1 or 2). This sampling of participants will be separate from the aims 1 and 2 samples. The focus for this aim will be on participants within 4 to 6 weeks of their recommended annual screening LDCT. Participants will be eligible for annual screening by CMS or USPSTF guidelines, English speaking, and due for their annual LDCT scan within 4 to 6 weeks. Participants who do not agree or consent to participate in the reminder evaluation interviews or are not eligible for annual screening (aged out of screening age, >15 years since quitting smoking, or competing comorbidities) will be excluded from this study.

### LCS-Eligible Participant Recruitment

Participants eligible for aim 3 will be due for their annual screening within 4-6 weeks. These participants will be identified in the database that is used to clinically manage the screening program at the University of Colorado Hospital. Recruitment strategies and informed consent procedures will mirror those used for aim 2.

### LCS-Eligible Participant Interview Guide and Procedures

Evaluation interviews will be conducted on the Zoom platform, with participants shown each reminder message content and imagery combination during the interview. The interview guide will include both open- and closed-ended questions and is based on prior research work in adherence for mental health appointments [53]. A total of 3 questions will focus on the understanding of the message content, 3 questions will ask about the message appeal (likes and dislikes), and 1 question will ask about how wording or content could be improved (Multimedia Appendix 1 [30-43]). Interview procedures and participant compensation will be parallel to those used in the aim 2 photovoice interviews. Participants will be asked to evaluate approximately 20 to 25 message candidates, 7 to 8 for each message-type platform (letter, postcard, and SMS text messaging). The interviews are expected to last approximately 60 minutes, and participants will receive US $60 as compensation on the completion of the interview.

### LCS-Eligible Participant Sample Size

Rapid, iterative feedback and refinement of messages will be completed in cycles comprised of 3 participants. It is expected that usability testing will consist of 3 to 4 iterative cycles, with a total of 9 to 12 LCS participants.

### LCS-Eligible Participant Evaluation Analysis Plan

The participant evaluation will use qualitative and quantitative data collected from the same survey instrument and will therefore be completed using the same procedures as in the aim 2 analysis. Quantitative and qualitative data will be analyzed separately and then merged to make final inferences and recommendations for further reminder message refinement. It is expected that the data types will corroborate one another, with higher-rated messages and visuals containing positive and encouraging themes and lower-rated messages associated with themes about confusion or misunderstanding of the message. The matrix design will also offer the opportunity for the qualitative data to explain the quantitative ratings and highlight the specific aspects the participants did or did not like about high- or low-rated messages.

### Refinement and Determination of Final Reminder Messages for Further Testing

**Figure 2:**
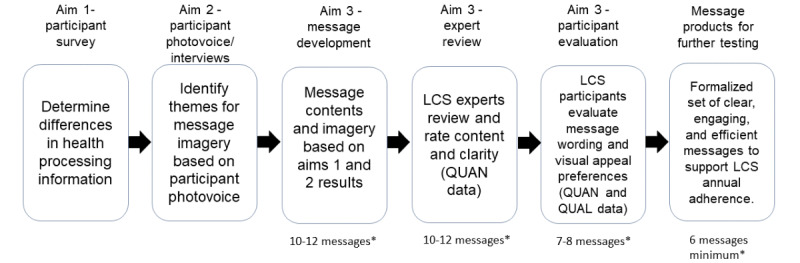
Summary of the message development process. *Per message-type (letter, postcard, and SMS text messaging). LCS: lung cancer screening; QUAL: qualitative data; QUAN: quantitative data.

The determination of the final reminder messages for each reminder type and matched visual picture will be driven by 3 main criteria: understanding, appeal rating, and anticipated impact. Difficulties in understanding the core message or with specific words will be used to refine the wording. Final message determination will consider results from both the usability expert survey and participant interviews by comparing the concordance or discordance between overall message ratings. Study team consensus will determine the final message set. The entire message development process is illustrated in Figure 2. The study team expects to have a minimum of 6 messages per communication type ready for robust testing in busy clinical settings.

### Ethics Approval, Informed Consent, and Participation

This study was approved by the Colorado Multiple Institutional Review Board (COMIRB #21-3881) with full waivers of informed consent and Health Insurance Portability and Accountability Act of 1996 authorization to use LCS clinical databases and electronic health records to identify potential participants. In addition, a waiver of written documents for informed consent was obtained to allow for the ease of the consent process in REDCap. Participants in all aims will agree to the contents of an informational consent form containing details about the study purpose, procedures, confidentiality, and use of data before the initiation of any study procedures.

## Results

The estimated milestones for study completion include (1) six months for setup (institutional review board approval, initiating procedures at accrual sites, updating the literature review for survey and interview guide development), which was completed in July 2022; (2) nine months for aim 1 survey recruitment, data collection, and analysis (expected completion: March 2023); (3) four months for aim 2 recruitment, data collection, and analysis (expected completion: March 2023); and (4) three months for aim 3 development of reminder message content and imagery combinations, recruitment, data collection, analysis, and refinement of final message candidates (expected completion: June 2023).

## Discussion

### Expected Outcomes

This project proposes a novel approach to facilitate adherence to annual LCS through the development of reminder messages that embrace content and imagery representative of the target population directly in the design process. Developing effective strategies to increase LCS adherence is instrumental in achieving optimal LCS outcomes at individual and population health levels. Engaging participants in yearly screening will ultimately increase the number of early-stage lung cancers detected, leading to improved outcomes and quality of life. Interventions aimed at addressing this critical need must be designed with the unique LCS population and context in mind and eliminate stigmatizing and biased content and imagery.

This research will develop message content based on health information processing theory conceptualized with the C-SHIP model, which recognizes health protective behavior decisions are the result of an interaction between the individual and their environment [22]. C-SHIP provides a broad framework to describe the characteristics of LCS-eligible individuals and constructs that may influence decisions to adhere to annual screening and to explore options for message targeting and tailoring. This project also aims to pinpoint imagery that is visually appealing and engaging in the LCS-eligible population. Imagery is often considered a significant component of health messaging and is even more important for individuals with low health literacy [54]. Specific to LCS, previous qualitative research in individuals at high risk for lung cancer found that images with cigarettes and lungs are ineffective in informational campaigns [55], highlighting the importance of identifying appropriate imagery for LCS communication. Finally, the resulting reminder messages will be iteratively tested with both LCS experts and participants to refine the message accuracy, understandability, and value of the content and imagery combinations.

### Limitations

Although this approach will leverage the strengths of participatory research and user-centered design methods, there are limitations that must be acknowledged using the available settings and resources. First, we will not be able to assess social cognitive theory for all LCS populations that experience health disparities and could benefit from message targeting and tailoring (eg, lesbian, gay, bisexual, transgender, queer or questioning persons or those with mental and substance disorders). Second, Colorado’s population lags the US population with regard to African American representation but exceeds the national average of individuals with Hispanic ancestry [56]. These demographic differences may compromise the generalizability of the initial development and testing of the message combinations. Finally, the efficacy and generalizability of these messages to clinical and health care settings are unknown. Despite these limitations, the design choices are appropriate to stimulate collaborative development of interventions directed specifically at improving LCS annual adherence and to highlight important areas of the future to strengthen the healthy equity reach and future efficacy, effectiveness, and implementation of the intervention.

## Appendix

Multimedia Appendix 1 Key questions and instructions for aims 1, 2, and 3.

Multimedia Appendix 2 Peer review report by National Cancer Institute Special Emphasis Panel - National Cancer Institute - NCI Predoc to Postdoc Fellow Transition Award (F99/K00) (National Institutes of Health, USA).

## Abbreviations

CDC: Centers for Disease Control and Prevention
CMS: Centers for Medicare and Medicaid Services
C-SHIP: Cognitive-Social Health Information Processing
LCS: lung cancer screening
LDCT: low-dose computed tomography
NIH: National Institutes of Health
REDCap: Research Electronic Data Capture
SatMDT: Step approach to Message Design and Testing
USPSTF: United States Preventive Services Task Force
