# Supplementary material for: Using User-Centered Design to Facilitate Adherence to Annual Lung Cancer Screening: Protocol for a Mixed Methods Study for Intervention Development
Source: JMIR Res Protoc. 2023 Apr 14;12:e46657. doi: 10.2196/46657 (PMC10162485; doi:10.2196/46657)
Supplement: Multimedia Appendix 1 [file resprot_v12i1e46657_app1.docx]

**Multimedia Appendix 2**

Key questions and instructions for aim 1, 2, and 3.

**Aim 1: Quantitative survey content**

| **Survey section and relevant** **LCS^a,b^ construct** | **Measure or Scale** |
| --- | --- |
| **Health-relevant encodings (C-SHIP^c^ domain)** | |
| Lung cancer risk perception | LCS Health Belief scales--perceived risks scale [30] |
| Functional well-being/health | In general, how would you rate your health?—Adapted from HINTS^d^ cycle 5 [31] |
| Comorbidities/life expectancy | Health Perceptions Questionnaire–health worry/concern scale [32] |
| **Health beliefs and expectancies (C-SHIP domain)** | |

| Self-efficacy | LCS Health Belief Scales—self-efficacy scale [30] |
| --- | --- |
| Beliefs about LCS | Self-Regulatory Questionnaire for Lung Cancer Screening—behavioral response subscale [33] |
| Family history of lung cancer | Have any of your parents, siblings, or children ever been diagnosed with lung cancer?—written for survey |
| LCS-specific knowledge | LCS Knowledge Survey [34] |
| **Affects (emotions; C-SHIP domain)** | |
| Lung cancer stigma | Cataldo Lung Cancer Stigma Scale—smoking subscale [35] |
| Fatalism | Revised Powe Fatalism Inventory [36] |
| Worry about cancer diagnosis | Self-Regulatory Questionnaire for Lung Cancer Screening—emotional representation scale [33] |
| **Health goals and values (C-SHIP domain)** | |
| Provider recommendation | Did a doctor, nurse, or other health professional recommend you have a low-dose CT^e^ for lung cancer screening?—written for survey |
| LCS risks and benefits | LCS Health Belief Scales—perceived risk & benefit scales [30] |
| Overall belief in healthy lifestyle | Health Behavior Scale for Cancer Patients [37] |
| Importance of early detection | Self-Regulatory Questionnaire for Lung Cancer Screening—illness coherence subscale [33] |
| Consequence of a lung cancer diagnosis | Self-Regulatory Questionnaire for Lung Cancer Screening—consequences subscale [33] |
| **Self-regulatory competencies (C-SHIP domain)** | |
| Barriers to continued screening | LCS Health Belief Scales—perceived barriers scale [30] |
| Health info seeking behavior | Medical Minimizer Maximizer Scale 1 [38] |
| Control over cancer diagnosis | Self-Regulatory Questionnaire for Lung Cancer Screening—personal control and treatment control subscales [33] |
| Health literacy (subjective) | BRIEF Health Literacy Scale [39] |
| Health numeracy (subjective) | Subjective Numeracy Scale [40] |
| **Previous LCS experience** | |
| Screening experience (SDM^f^, LDCT^g^, results, and intent) | - Regular source of health care (adapted from HINTS/cycle 5) [31] - Did a clinician recommend LCS, explain screening is yearly, explain risks and benefits (SDM)—written for survey - SDM questions—9-item Shared Decision-Making Questionnaire [41] - LDCT experience (location, time waited, concerns addressed, stigma experienced, overall satisfaction)—written for survey - How long waited for results—written for survey - Emotions while waiting—Adapted from Woolen, et al. [42] - Intent to adhere to annual screening—written for survey |
| **Reminder message preferences** | |
| Modality and timing | - Modality type (reminder effectiveness of call to home phone, call to cell phone, detailed letter, basic postcard, email, text, and patient portal)—written for survey - Modality preference (type of reminder used matters, all reminder should be same type)—written for survey - Timing (before/ or after appointment, remind or upcoming appointment or prompt to make appointment)—written for survey |
| **Respondent information** | |
| Demographics, clinical characteristics, smoking history | All questions adapted from HINTS cycle 5 [31]   - Age - Gender identity - Occupational status - Health insurance - Marital status - Education - Race - Ethnicity - Income - Comorbid conditions - Cigarette smoking history   1. Current smoking status   2. Starting age   3. Quit age (if applicable)   4. Average number of cigarettes smoked per day |

^a^LCS: lung cancer screening.

^b^Lung cancer screening constructs relevant to each C-SHIP domain and scale/measure used to measure each construct.

^c^C-SHIP: Cognitive-Social Health Information Processing Model.

^d^HINTS: Health Information National Trends Survey

^e^CT: computed tomography.

^f^SDM: shared decision-making

^g^LDCT:low-dose computed tomography

**Aim 2: Directions provided to participants for photovoice activity**

*Directions are provided in the REDCap database after the informational consent is acknowledged.*

The first step in this research project is to find three photos that represent what lung cancer screening means to you. You can take photos yourself or find pictures using Google Images (www.google.com/imghp) or other places on the internet.

Please spend a little time thinking about what pictures would best represent lung cancer screening. Some things you might want to consider include:

- Why did you decide to be screened for lung cancer?

- What motivated you to be screened for lung cancer?

- What aspects in your life helped you decide to be screened for lung cancer?

- How did being screened for lung cancer make you feel about your life and health?

- What do you think about continuing to participate in lung cancer screening?

If helpful, you can write down words or ideas that will help you find or capture the right pictures that represent lung cancer screening to you. It is your choice if you would like to take photos yourself or find pictures on the internet using Google Images ([www.google.com/imghp](http://www.google.com/imghp)).

After you have taken or found your pictures please return to this study webpage to attach or provide a link to each picture you selected to represent lung cancer screening by clicking on the 'Save & Return Later' button at the bottom of this page. You can return to this page at any time by clicking on the link for this database available in your email.

After you provide the information for the three pictures you have chosen for this study you will contacted to set up the interview to take place on the Zoom platform.

Any information you provide here will be kept strictly confidential and will be used only for research purposes.

**Aim 2:Photovoice interview questions adapted from the SHOWeD technique [43]**

| The SHOWeD Technique | Adapted Interview Question |
| --- | --- |
| What do you See here? | To begin, please describe what you see when you look at this picture? What about what you feel? |
| What's really Happening here? | Tell me about what you thinking is happening in this photo. |
| How does this relate to Our lives? | What does this picture mean to you? |
| Why does this problem, concern, or strength Exist? | Why did you select this picture to represent lung cancer screening? |
| What can we Do about it? | How could this help support lung cancer screening? |

**Aim 2: Semi-structured interview guide**

| **Introduction** | - Thank you for agreeing to participate in this interview. - The goal of this research project is to identify pictures and imagery engaging to individuals that are eligible for lung cancer screening. - Lessons learned about imagery from these interviews will be used to help develop reminder messages specifically for lung cancer screening. - We are interested in hearing about the images you chose for this project. - Do you have questions before we begin the interview recording? - Can you please verbalize that you agree with audio recording of this interview? |
| --- | --- |
| **REPEAT QUESTIONS 1-7 FOR ALL UPLOADED PHOTOS (n = 3)** | |
| **Meaning of photo**  **SHOWeD technique (See, Happening, relate to Our lives)** | 1. To begin, please describe what you see when you look at this picture? What about what you feel?    1. Probe: Give me a summary of what you see 2. Tell me about what you think is happening in this photo. 3. What does this picture mean to you?    1. Probe: Describe what about this photo represents in your life.    2. Probe: What words would you use to describe the meaning of this photo? |
| **Representation of lung cancer screening**  **SHOWeD technique (Why and Do)** | 1. Why did you select this picture to represent lung cancer screening?    1. Probe: What about this photo makes you feel…….    2. Probe: What aspects of your life have influenced how this photo represents lung cancer screening to you?   5) How could this help support lung cancer screening? |
| **Likes, dislikes, and overall rating of photo** | 6) What do you like most and least about this picture?   1. Probe: What is the one thing you like most about this picture? 2. Probe: What is the one thing you like least about this picture? 3. Probe: Tell me more about what you like (or dislike) about that part of the photo.   7) On a scale of 0-10, where 0 is ‘I don’t like this photo’ and 10 is ‘I really like this photo,’ what rating do you give this photo? |
| **Other imagery** | 8) Are there any other pictures that you might have wanted to select to represent lung cancer screening to you?   1. Probe: If yes, can you tell me more about that? |
| **SHOW IMAGES WITH LUNGS/ CIGARETTES/CT SCANNERS FOR QUESTIONS 9 - 11** | |
| **Perceptions of common images used for lung cancer screening communication** | 9) What are your thoughts about these images? How do they make you feel?   1. Probe: What is the one thing you like most about this picture? 2. Probe: What is the one thing you like least about this picture?   10) Tell me about what these images say about lung cancer screening to you.   - 1. Probe: What about this photo makes you feel…….   11) On a scale of 0-10, where 0 is ‘I don’t like this photo’ and 10 is ‘I really like this photo,’ what rating do you give this photo? |
| **Final question** | 12) Is there anything else you would like to tell me about images and lung cancer screening? |
| **Closing** | Thank you very much for answering questions about the pictures you chose for this study. Your input and time are greatly appreciated!  Have a wonderful day! |

**Aim 3: First round of iterative evaluation with lung cancer screening experts (pulmonologists, radiologists, medical oncologists, primary care providers, advanced practice providers, and implementation scientists).**

*Lung cancer screening experts will be asked to evaluate approximately 30 – 36 reminder message combinations with varying content and imagery, including 10 – 12 messages for each communication platform (postcard, letter, SMS). A mock-up of each message combination with an associated clinical vignette for targeting or tailoring information (if applicable) will be displayed in a REDCap database and experts will be asked to evaluate each combination using a seven-point quality Likert scale where 1 is ‘Very poor’ and 7 is ‘Very good.’*

Experts will rate the following categories for each content and imagery combination:

1) relevance of the message content to lung cancer screening adherence,

2) readability of the message content for lung cancer screening participants,

3) accuracy of the message content for lung cancer screening participants,

4) intent of the message content (conveying screening benefit),

5) memorability of the visual appeal for lung cancer screening adherence, and

6) overall rating of the message content and visual imagery combination.

**Aim 3: Second round of iterative evaluation with lung cancer screening participants that are due for their annual CT within 4-6 weeks.**

*The second round of iterative evaluation will be completed with lung cancer screening participants that are close to their annual screening CT through mixed-methods key informant interviews. Interview participants will be asked the following seven questions for approximately 20 – 25 content and imagery combinations (7 – 8 message combinations for each platform (letter, postcard, SMS)).*

1. If you had to explain what this message says, what would you say?
2. Do you understand every word in this message?
3. Which word(s) do you not understand? (asked if applicable)
4. Would you please explain what you like about this message/picture?
5. Would you please explain what you do not like about this message/picture?
6. Imagine you were sent this message to remind you to make an appointment for your upcoming lung cancer screening. From a scale of 0 to 10, where 0 is ‘I don’t like this message’ and 10 is ‘I really like this message’, what score would you give this message?
7. Do you have any suggestions on how to improve the content of this message?
