# Supplementary material for: Using User-Centered Design to Facilitate Adherence to Annual Lung Cancer Screening: Protocol for a Mixed Methods Study for Intervention Development
Source: JMIR Res Protoc. 2023 Apr 14;12:e46657. doi: 10.2196/46657 (PMC10162485; doi:10.2196/46657)
Supplement: Multimedia Appendix 2 [file resprot_v12i1e46657_app2.pdf]

PROGRAM CONTACT:
Mariam Eljanne
240-276-7607
eljannem@mail.nih.gov

Release Date: 04/25/2021
Revised Date:

Application Number: 1 F99 CA264409-01

HIRSCH,ERIN
University of Colorado Denver
12401 East 17th Avenue
Aurora, CO 800452548

Review Group:	ZCA1 SRB-H (M1)<br>National Cancer Institute Special Emphasis Panel<br>NCI Predoc to Postdoc Fellow Transition Award (F99/K00)
Meeting Date:	03/23/2021
Council:	MAY 2021 *PCC:* X6TR
Requested Start:	08/01/2021

Project Title:	Optimizing adherence to lung cancer screening: Applying theory and implementation science to participant engagement
	Requested:
Sponsor:	
Department:	21419--CSPH-Bio Info General O
Organization:	UNIVERSITY OF COLORADO DENVER
City, State:	AURORA COLORADO
SRG Action:	Impact Score:13
Next Steps:	Visit https://grants.nih.gov/grants/next_steps.htm
Human Subjects:	30-Human subjects involved - Certified, no SRG concerns
Animal Subjects:	10-No live vertebrate animals involved for competing appl.
Gender:	1A-Both genders, scientifically acceptable
Minority:	1A-Minorities and non-minorities, scientifically acceptable
Age:	3A-No children included, scientifically acceptable

1F99CA264409-01 Hirsch, Erin

RESUME AND SUMMARY OF DISCUSSION: The fellowship candidate, Erin Hirsch, proposes training in optimizing early detection of lung cancer by facilitating adherence to annual lung cancer screening (LCS). The goal of her dissertation research training is to use a theory-informed, multiphase, mixed-methods approach to develop a set of clear and engaging reminder messages to support LCS annual adherence. The highly motivated candidate has a stellar academic record and robust productivity, with several relevant research articles (two as first author) and another submitted. Her highly focused career goals are firmly supported by substantial prior research experience as a clinical research coordinator, including work on expanding use of lung cancer screening. The letters of reference attest to her strong commitment and potential to become an independent and productive researcher. Her highly qualified sponsors, Dr. Jamie Studts and Dr. Christina Studts, have complementary expertise and adequate research funds to support the candidate's F99 Phase, and both have previously successfully mentored multiple graduate students, fellows and junior faculty. The candidate will be further supported by the three collaborators, Drs. Glasgow, Thomas and Scherer, to enhance her training and progress toward the K00 Phase. The excellent institutional environment at the University of Colorado Medical School includes the Qualitative and Mixed Methods Research Core and the Dissemination and Implementation Science Program to uniquely position the candidate for success. The well-described training plan includes a feasible time frame and clear milestones. Notably her research is synergistic with the sponsor's but sufficiently distinct from sponsor's and consistent with candidate's stage of development and research career objectives. The research plan and training plan are well aligned, with well-defined steps from the F99 Phase to the K00 Phase. For the K00 phase, the candidate will focus on hybrid and pragmatic study designs and measures to simultaneously assess intervention effectiveness and implementation outcomes. The candidate will gain additional training in dissemination and implementation science and methodology to ultimately develop and implement effective, low-burden interventions to improve LCS adherence, maximize screening benefit, and reduce lung cancer mortality. Potential minor weaknesses of the application relate to inadequate discussion of representativeness of survey participants and non-participants and the external validity of the findings. Also, it is unclear whether the candidate considered evaluating reminder message content from previous studies to inform the presently proposed design. Another consideration is that the K00 research plan appears to lack a comparison group that is not exposed to the reminder intervention. Overall, the candidate is outstanding, and the application is expected to have a high impact in enhancing the candidate's potential for and commitment to a successful independent career in cancer research.

DESCRIPTION (provided by applicant): Lung cancer is the leading cause of cancer death in the US, with most lung cancer diagnosed at advanced stages. Low-dose CT screening of high-risk individuals is the only evidence-based tool available to diagnose lung cancer at an early stage when curable treatment options exist. The promising mortality reduction benefit of lung cancer screening (LCS) is presently overwhelmingly unfulfilled due to suboptimal population-based adherence to annual screening guidelines. Outside of research settings, adherence to annual LCS is less than half of the rates observed in the practice-changing, landmark clinical trials. There is an urgent need for effective and feasible interventions to improve LCS adherence and achieve optimal individual and population health benefits. Research during the F99 phase will leverage an innovative, multi-phase, mixed methods design to describe how LCS patients process health information regarding health protective behavior and utilize this information to develop and evaluate reminder messages with individuals eligible for LCS. Using a four-step process, data collected in a survey of LCS program participants (step 1) will inform the development of a pool of candidate reminder messages (step 2). In the final two steps, the reminder messages will be evaluated by LCS experts (step 3) and further evaluated and refined with LCS program participants (step 4) using mixed methods, including surveys and interviews. Differences between health information processing constructs by demographic or clinical characteristics found to be significant from step 1 will be the focus for message targeting (group level) and/or tailoring

(individual level). At the conclusion of the F99 phase, I will have a formalized set of clear, engaging, and efficient messages to support LCS annual adherence, ready to be evaluated in a clinical setting. Research proposed in the K00 phase will focus on gaining real-world experience with hybrid effectiveness implementation study designs and pragmatic outcome measures to simultaneously assess effectiveness and implementation outcomes. Specifically, I propose a mentored pre-post type 1 hybrid effectiveness- implementation trial, within a parent trial, that will allow concurrent assessment of effectiveness and implementation outcomes of the F99 reminder strategy in real-world LCS programs. Effectiveness will be tested using a pre-post design, comparing percent screening participants adherent to annual screening guidelines before and after implementation of the reminder strategy. Secondary implementation outcomes (i.e., acceptability and feasibility) will be assessed with surveys and key informant interviews of personnel involved in the reminder system implementation (e.g., LCS program directors, navigators, coordinators). This body of research will prepare me for a career as an independent cancer-focused intervention scientist with expertise in the development and implementation of effective, low-burden interventions aimed at improving LCS adherence, maximizing screening benefit, and reducing lung cancer mortality.

PUBLIC HEALTH RELEVANCE: Lung cancer screening efficacy is highly dependent on adherence to recommended guidelines, with annual screening indicated for approximately 90% of all individuals who receive baseline scans. Initial reports of real- world screening implementation show adherence to annual screening guidelines are less than 50%, demonstrating a critical need for improvement to optimize the individual and population health benefits of lung cancer screening. I aim to address the urgent need for low-cost, feasible, and effective interventions focused on facilitating lung cancer screening adherence by developing and implementing a set of clear and engaging reminder messages that target and tailor information specific to lung cancer screening participants.

CRITIQUE: The written critiques of individual reviewers are provided in essentially unedited form in this section. Please note that critiques and criteria scores, prepared prior to the review meeting, may not have been revised following discussions at the meeting. The "Resume and Summary of Discussion" section summarizes the final opinions of the review committee.

CRITIQUE 1

Fellowship Applicant:	1
Sponsors, Collaborators, and Consultants:	1
Research Training Plan:	3
Training Potential:	1
Institutional Environment & Commitment to Training:	1

Overall Impact/Merit: This is an outstanding candidate with a strong background who provided an excellent description of her career development/training and research plans to address an important problem. She has identified an outstanding sponsor/sponsorship team who are ideally suited to help her in this work. A minor limitation is the lack of prior collaboration with her team due to the recent departure of her primary mentor, but the suitability of her sponsor and the clear engagement in the candidate’s proposal and current and ongoing work temper this limitation. Minor limitations in the proposed research are also addressable.

1. Fellowship Applicant
Strengths
- Candidate has strong commitment to career focused on implementation research to identify effective and low burden interventions to improve lung cancer screening
- Substantial prior research experience as a clinical research coordinator, including work focused on expanding use of lung cancer screening

- Three papers published (two as first author that are directly related to proposed research); another submitted
- Strong candidate for career as an independent researcher
- Outstanding letters suggest a strong commitment to and high potential for a career as an independent researcher

Weaknesses

- None noted.

2. Sponsors, Collaborators, and Consultants

Strengths

- Although candidate's prior mentor left academic practice, she quickly identified an outstanding mentor and mentorship team and together they have developed a well-planned dissertation research program and plans for transition to postdoctoral training
- Candidate began collaborating with her mentor even before his arrival at the University of Colorado
- Expertise of sponsor and co-sponsor is complementary and matches the candidate's interests
- Dr. J Studts (sponsor) has expertise in behavioral aspects of lung cancer early detection and survivorship
- Dr. C Studts (co-sponsor) has expertise in dissemination and implementation science
- Dr. Scherer has expertise in medical decision making and will assist with reminder messaging
- Dr. Glasgow has expertise in implementation science
- Dr. Thomas directs the lung cancer screening program and will provide clinical expertise
- Sponsor has very strong track record in mentoring, mentorship awards
- Team structure is well justified
- Applicant has described a strong plan for identifying a mentor and institution for the K00 phase of the award.
- Sponsor team members were very engaged in F99/K00 process
- There are adequate research funds to support the applicant's proposed research and training

Weaknesses

- New relationship with sponsors and co-sponsors because prior mentor left, but candidate has done an extraordinary job at engaging a new primary mentor and mentorship team that are extremely well suited for the work, turning this into a strength.

3. Research Training Plan

Strengths

- Research addresses important questions, well grounded in theory, and is well integrated with training plan
- Both phases of research well designed/described
- F99 phase leads directly to plans for K00 phase
- Research is synergistic with sponsor's but sufficiently distinct from sponsor's and consistent with applicant's stage of development and research career objectives
- Time frame is feasible to accomplish proposed dissertation research training
- Candidate provides a clear outline of feasible research milestones

Weaknesses

- Lack of discussion about representativeness of likely survey participants versus non-participants and how nonresponse and focus on individuals at one lung cancer screening program may affect external validity of findings
- For K00 phase, pre-post design is reasonable given the goal of a pragmatic trial in a real-world setting in which all eligible participants receive the intervention. However, the lack of a

comparison group is a limitation, as rates of follow up screening could be changing over time. The study would be enhanced tremendously if they identified a comparison group that is not exposed to the reminder intervention (e.g., if in Colorado, perhaps making use of the Colorado all payer claims data would allow this)

4. Training Potential/Career Development Plan
Strengths
- F99 phase has high potential to provide a strong foundation for K00 phase
- Additional course work proposed in F99 and K00 is appropriate for research goals.
- Training plan designed to fill gaps and provide key skills for transition to career development phase
- Milestones for F99 phase are outlined clearly
- Sponsor and cosponsor have clearly described plans for supervision during the training period

Weaknesses
- None noted.

5. Institutional Environment & Commitment to Training
Strengths
- Outstanding environment
- Clear description of research facilities, resources, and many training opportunities that will increase candidate's likelihood of success
- Strong institutional commitment to fostering the applicant's mentored training in the F99 phase (including additional funds from the University of Colorado Cancer Center (UCCC) Deputy Directory Dr. Cathy Bradly to demonstrate very strong support for the candidate.
- Excellent description of needs for transition to K00 phase and good ideas about possible opportunities that will support candidate and match the research program anticipated in that phase.

Weaknesses
- None noted.

Protections for Human Subjects: Acceptable Risks and Adequate Protections. Appropriate protection

Data and Safety Monitoring Plan (Applicable for Clinical Trials Only): Acceptable. Very brief description, could benefit from more details

Inclusion Plans
- Sex/Gender: Distribution justified scientifically
- Race/Ethnicity: Distribution justified scientifically
- For NIH-Defined Phase III trials, Plans for valid design and analysis: Scientifically acceptable
- Inclusion/Exclusion Based on Age: Distribution justified scientifically

Biohazards: Not Applicable (No Biohazards)

Resubmission: Not Applicable

Renewal: Not Allowed

Training in the Responsible Conduct of Research: Acceptable
Comments on Format (Required):
- Excellent description

Comments on Subject Matter (Required):
- Excellent description

Comments on Faculty Participation (Required):
- Excellent description

Comments on Duration (Required):
- Excellent description

Comments on Frequency (Required):
- Excellent description

Select Agents: Not Applicable (No Select Agents)

Resource Sharing Plans: Not Applicable (No Relevant Resources)

Budget and Period of Support: Recommend as Requested.

CRITIQUE 2

Fellowship Applicant:	2
Sponsors, Collaborators, and Consultants:	1
Research Training Plan:	1
Training Potential:	2
Institutional Environment & Commitment to Training:	1

Overall Impact/Merit: This is a well-written application from an excellent candidate. The training plan is well developed and, in conjunction with the research project, will help Ms. Hirsch develop new skills and expertise and enhance her existing skills. The research project addresses an important question and is of high scientific quality. The sponsors and collaborators, as well as the environment and available resources for the proposed research project and training are excellent. Overall, the fellowship is expected to provide Ms. Hirsch with the skills and knowledge to develop, design and implement effective intervention strategies for early detection of cancer and to significantly enhance her potential to become a successful independent cancer researcher.

1. Fellowship Applicant
Strengths
- The applicant, Ms. Hirsch, has an outstanding academic record and research background. She has shown a clear commitment to a career as an independent researcher specifically focused on the development, design and implementation of effective intervention strategies for early detection of cancer.
- Strong letters of reference that confirm Ms. Hirsch’s strong commitment to and high potential for becoming an independent researcher.

Weaknesses
- None noted.

2. Sponsors, Collaborators, and Consultants
Strengths

- The two proposed sponsors, Drs. J. Studts and C. Studts, have complementary expertise and skills, and are well-positioned to successfully mentor and train Ms. Hirsch. Both have previously successfully mentored multiple graduate students, fellows and junior faculty. Ms. Hirsch will further be supported by the three collaborators, Drs. Glasgow, Thomas and Scherer, and together the team offers expertise in every area targeted in her training plan.
- Adequate funds are available to support Mr. Hirsch's proposed project and training during the F99 phase. The University of Colorado Cancer Center will provide an additional $10,000 in support.
- An appropriate set of characteristics for the K00 institution as well as the K00 mentor and environment are described.

Weaknesses

- None noted.

3. Research Training Plan

Strengths

- The proposed research project is outstanding. The project addresses a critical issue, suboptimal adherence to annual lung cancer screening guidelines, and is well integrated with the proposed training plan. The project will leverage a multi-phase, mixed methods design to describe how lung cancer screening patients process information regarding health protective behavior and utilize this information to develop and evaluate reminder messages with individuals eligible for lung cancer screening. The project is highly relevant to Ms. Hirsch's research career objectives.
- The proposed research project is sufficiently distinct from her sponsors' funded research.
- The proposed timeline is feasible.
- The application includes a clear description of research and training planned during the K00 phase. Research proposed for the K00 phase will focus on gaining real-world experience with hybrid effectiveness implementation study designs and pragmatic outcome measures to simultaneously assess effectiveness and implementation outcomes. The applicant proposes to conduct a mentored pre-post type 1 hybrid effectiveness implementation trial, within a parent trial, that will allow concurrent assessment of effectiveness and implementation outcomes of reminder strategy developed in the F99 phase in real-world lang cancer screening programs.

Weaknesses

- None noted.

4. Training Potential/Career Development Plan

Strengths

- The proposed research project and training plan are well-developed and well-reasoned. They will allow Ms. Hirsch to gain experience in qualitative and mixed-methods research, learn health behavior theory for application to user-centered design, and to further enhance her expertise in dissemination and implementation science. Training activities planned include individualized tutorials by the sponsors as well as formal course work.
- Career, scientific and professional milestones are clearly described. The application includes detailed plans for mentoring as well as for monitoring and evaluating Ms. Hirsch's research and career development progress.

Weaknesses

- None noted.

5. Institutional Environment & Commitment to Training

Strengths

- The facilities and resources required for the proposed research project are all available.

- A particular strength is the presence of the Qualitative & Mixed Methods Research Core, which is dedicated to engaging and building knowledge in rigorous qualitative and mixed methods research across the University of Colorado Anschutz Medical Campus, and the Dissemination and Implementation (D&I) Science Program which is led by collaborator Dr. Glasgow and includes co-sponsor Dr. C. Studts as a core team member.
- The environment at the University of Colorado Anschutz Medical Campus is excellent for the proposed research project and training. In addition, there is a strong collaborative network of specialists in lung cancer screening in the Greater Denver - Aurora area that Ms. Hirsch will also be able to utilize for advice and collaborations.
- The applicant plans to focus her postdoctoral research on developing advanced skills in D&I science, hybrid study design, and pragmatic research outcomes.
- The application includes detailed information on training goals/objectives and planned training activities for both the F99 and K00 phases. The facilities, resources and training opportunities for the K00 phase adequately match the research program anticipated during that phase.

Weaknesses

- None noted.

Protections for Human Subjects: Acceptable Risks and Adequate Protections.

Data and Safety Monitoring Plan (Applicable for Clinical Trials Only): Acceptable.

Inclusion Plans

- Sex/Gender: Distribution justified scientifically
- Race/Ethnicity: Distribution justified scientifically
- For NIH-Defined Phase III trials, Plans for valid design and analysis:
- Inclusion/Exclusion Based on Age: Distribution justified scientifically
- Comment: The planned recruitment numbers are based on the demographics of the subjects that screened have been for lung cancer through the University of Colorado Hospital screening program. It is unclear whether there is additional outreach to recruit minority study participants.

Biohazards: Not Applicable (No Biohazards)

Resubmission: Not Applicable

Renewal: Not Allowed

Training in the Responsible Conduct of Research: Acceptable

Comments on Format (Required):

- Adequate: face-to-face lectures, group discussions, and online (CITI) courses

Comments on Subject Matter (Required):

- Comprehensive. In addition to standard topics, the applicant plans to also specifically address challenges related to doing research on lung cancer screening in under-served communities.

Comments on Faculty Participation (Required):

- Sessions and lectures are led by faculty members. In addition, subjects like authorship, protection of human subjects, conflict of interest will also be discussed with the sponsors.

Comments on Duration (Required):

- More than eight contact hours in total

Comments on Frequency (Required):

- Adequate

Select Agents: Not Applicable (No Select Agents)

Resource Sharing Plans: Acceptable. The proposed resource sharing plan is reasonable.

Budget and Period of Support: Recommend as Requested.

CRITIQUE 3

Fellowship Applicant:	3
Sponsors, Collaborators, and Consultants:	1
Research Training Plan:	3
Training Potential:	3
Institutional Environment & Commitment to Training:	2

Overall Impact/Merit: The application describes a detailed plan to develop informed annual lung cancer screening decisions in lapsed individuals. Candidate has developed a close, supportive mentored relationship with leaders in her field. The methods for the research plan and the contributions of mentors are well articulated. The proposed plan has a high chance of facilitating successful career development.

1. Fellowship Applicant

Strengths

- 3.9+ GPA in MS program in Clinical Science, U. Colorado.
- Establishing credentials in lung cancer screening adherence and interventions through publications and presentations. 10 or more years work experience in clinical studies of lung cancer.
- Strong support letters indicating high probability of success.

Weaknesses

- None noted.

2. Sponsors, Collaborators, and Consultants

Strengths

- Strong complimentary mentoring team of faculty in decision making, implementation science, behavioral interventions, and lung cancer screening expertise. Leaders in the field. Closely aligned with applicant's plans.
- Specific training skills that applicant seeks in collaboration with mentors well described.
- Clinical trial study design mentoring closely aligned with applicant goals.

Weaknesses

- None noted.

3. Research Training Plan

Strengths

- The need for the proposed project is justified.

- Research project in lapsed screened individuals build on mentor expertise in lung cancer interventions, and appears to be independently developed. Timeframe is feasible.
- Transition from developmental work in f99 to clinical intervention in K phase is appropriate.
- Proposed studies are feasible within timeframe.
- Time course of developing new skills fits well with background.

Weaknesses
- No apparent evaluation of reminder message content from previous studies and how that guides the proposal.

4. Training Potential/Career Development Plan
Strengths
- Plan details the transitions and skills needed at each step.
- Training goals and professional development described.

Weaknesses
- None noted.

5. Institutional Environment & Commitment to Training
Strengths
- Excellent interdisciplinary mentoring team that are leaders in respective fields and expertise in the specific goals of applicant.
- Applicant describes overall need and training-specific need for risk communication science in lung cancer screening, but training will allow for using skill set beyond lung screening.
- Home institution has resources to support proposed project.

Weaknesses
- None noted.

Protections for Human Subjects: Acceptable Risks and Adequate Protections. Minimal risk

Data and Safety Monitoring Plan (Applicable for Clinical Trials Only): Acceptable. Not a clinical trial, but DSMB will oversee.

Inclusion Plans
- Sex/Gender: Distribution justified scientifically
- Race/Ethnicity: Distribution justified scientifically
- For NIH-Defined Phase III trials, Plans for valid design and analysis:
- Inclusion/Exclusion Based on Age: Distribution justified scientifically
- Sample population includes all lung cancer screened individuals. Children are not eligible, as those outside the age range of 55-80 will be excluded.

Biohazards: Not Applicable (No Biohazards)

Resubmission: Not Applicable

Renewal: Not Allowed

Training in the Responsible Conduct of Research: Acceptable.

Comments on Format (Required):
- Formal training description described.

Comments on Subject Matter (Required):
- TA for formal coursework on ethics, CITI and regular contact with mentors.

Comments on Faculty Participation (Required):
- TA, other

Comments on Duration (Required):
- Continuous

Comments on Frequency (Required):
- Frequent multiple contacts

Select Agents: Not Applicable (No Select Agents)

Resource Sharing Plans: Acceptable

Budget and Period of Support: Recommend as Requested.

THE FOLLOWING SECTIONS WERE PREPARED BY THE SCIENTIFIC REVIEW OFFICER TO SUMMARIZE THE OUTCOME OF DISCUSSIONS OF THE REVIEW COMMITTEE, OR REVIEWERS' WRITTEN CRITIQUES, ON THE FOLLOWING ISSUES:

PROTECTION OF HUMAN SUBJECTS: ACCEPTABLE

INCLUSION OF WOMEN PLAN: ACCEPTABLE

INCLUSION OF MINORITIES PLAN: ACCEPTABLE. The planned recruitment numbers are based on the demographics of the subjects that screened have been for lung cancer through the University of Colorado Hospital screening program. It is unclear whether there is additional outreach to recruit minority study participants.

INCLUSION ACROSS THE LIFESPAN PLAN: ACCEPTABLE. Children will not be eligible for this study as lung cancer screening guidelines do not recommend screening anyone <55 years of age.

COMMITTEE BUDGET RECOMMENDATIONS: The budget was recommended as requested.

Footnotes for 1 F99 CA264409-01; PI Name: Hirsch, Erin

NIH has modified its policy regarding the receipt of resubmissions (amended applications).See Guide Notice NOT-OD-18-197 at https://grants.nih.gov/grants/guide/notice-files/NOT-OD-18-197.html. The impact/priority score is calculated after discussion of an application by averaging the overall scores (1-9) given by all voting reviewers on the committee and multiplying by 10. The criterion scores are submitted prior to the meeting by the individual reviewers assigned to an application, and are not discussed specifically at the review meeting or calculated into the overall impact score. Some applications also receive a percentile

ranking. For details on the review process, see
http://grants.nih.gov/grants/peer_review_process.htm#scoring.

MEETING ROSTER

National Cancer Institute Special Emphasis Panel
NATIONAL CANCER INSTITUTE
NCI Predoc to Postdoc Fellow Transition Award (F99/K00)
ZCA1 SRB-H (M1)
03/23/2021 - 03/24/2021

Notice of NIH Policy to All Applicants: Meeting rosters are provided for information purposes only. Applicant investigators and institutional officials must not communicate directly with study section members about an application before or after the review. Failure to observe this policy will create a serious breach of integrity in the peer review process, and may lead to actions outlined in NOT-OD-14-073 at https://grants.nih.gov/grants/guide/notice-files/NOT-OD-14-073.html and NOT-OD-15-106 at https://grants.nih.gov/grants/guide/notice-files/NOT-OD-15-106.html, including removal of the application from immediate review.

CHAIRPERSON(S)

YU, DAVID S, PHD, MD
ASSOCIATE PROFESSOR
DEPARTMENT OF RADIATION ONCOLOGY
WINSHIP CANCER INSTITUTE
EMORY UNIVERSITY SCHOOL OF MEDICINE
ATLANTA, GA 30322

MEMBERS

ALEXANDER, CAROLINE M, PHD
PROFESSOR
MCARDLE LABORATORY FOR CANCER RESEARCH
UNIVERSITY OF WISCONSIN-MADISON
MADISON, WI 53705

ANGEL, PEGGI M, PHD
ASSISTANT PROFESSOR
DEPARTMENT OF CELL AND MOLECULAR
PHARMACOLOGY & EXPERIMENTAL THERAPEUTICS
MEDICAL UNIVERSITY OF SOUTH CAROLINA
CHARLESTON, SC 29425

BIGATTI, SILVIA M, PHD
PROFESSOR
DEPARTMENT OF SOCIAL AND BEHAVIORAL SCIENCES
RICHARD M. FAIRBANKS SCHOOL OF PUBLIC HEALTH
INDIANA UNIVERSITY SCHOOL OF MEDICINE
INDIANAPOLIS, IN 46202

BODURKA, DIANE, MD
DEPARTMENT OF GYNECOLOGIC ONCOLOGY &
REPRODUCTIVE MEDICINE
THE UNIVERSITY OF TEXAS MD ANDERSON CANCER
CENTER
HOUSTON, TX 77030

BOUTON, AMY H, PHD
PROFESSOR
DEPARTMENT OF MICROBIOLOGY, IMMUNOLOGY
AND CANCER BIOLOGY
UNIVERSITY OF VIRGINIA SCHOOL OF MEDICINE
CHARLOTTESVILLE, VA 22908

CRUZ, C. RUSSELL Y., PHD, MD
ASSISTANT PROFESSOR OF PEDIATRICS
CHILDREN'S NATIONAL HOSPITAL
WASHINGTON, DC 20010

DIERGAARDE, BRENDA B, PHD
ASSOCIATE PROFESSOR OF HUMAN GENETICS
UPMC HILLMAN CANCER CENTER
GRADUATE SCHOOL OF PUBLIC HEALTH
UNIVERSITY OF PITTSBURGH
PITTSBURGH, PA 15213

FITZGERALD-BOCARSLY, PATRICIA, PHD
PROVOST, RUTGERS BIOMEDICAL AND HEALTH SCIENCES
PROFESSOR AND VICE-CHAIR
DEPARTMENT OF PATHOLOGY, IMMUNOLOGY &
LABORATORY MEDICINE
RUTGERS CANCER INSTITUTE OF NEW JERSEY
NEWARK, NJ 07101

GARIPPA, RALPH J, PHD
HEAD, GENE EDITING & SCREEN CORE FACILITY
DEPARTMENT OF CANCER BIOLOGY AND GENETICS
MEMORIAL SLOAN-KETTERING INSTITUTE AND CANCER
CTR
MEMORIAL SLOAN KETTERING INSTITUTE
NEW YORK, NY 10065

GEVAERT, OLIVIER, PHD
ASSISTANT PROFESSOR
DEPARTMENT OF MEDICINE
STANFORD CTR FOR BIOMEDICINE INFORMATICS RESEARCH
STANFORD UNIVERSITY
STANFORD, CA 94305

HAGENSEE, MICHAEL E, MD, PHD
PROFESSOR AND VICE CHAIR
DEPARTMENT OF MEDICINE
SECTION OF INFECTIOUS DISEASES
LOUISIANA STATE UNIVERSITY HEALTH SCIENCES CENTER
NEW ORLEANS, LA 70112

HEIN, DAVID W, PHD
PROFESSOR AND CHAIR
DEPARTMENT OF PHARMACOLOGY & TOXICOLOGY
UNIVERSITY OF LOUISVILLE HEALTH SCIENCES CENTER
LOUISVILLE, KY 40202

HICKS, STEPHANIE CARINNE, PHD
ASSISTANT PROFESSOR
DEPARTMENT OF BIOSTATISTICS
JOHNS HOPKINS BLOOMBERG SCHOOL OF PUBLIC HEALTH
BALTIMORE, MD 21205

HILAKIVI-CLARKE, LEENA A, PHD
PROFESSOR OF SCIENCE AND NUTRITION
SECTION LEADER OF WOMEN'S HEALTH
UNIVERSITY OF MINNESOTA HORMEL INSTITUTE
AUSTIN, MN 55912

HOUTMAN, JON C.D., PHD
ASSOCIATE PROFESSOR
DEPARTMENT OF MICROBIOLOGY
UNIVERSITY OF IOWA
IOWA CITY, IA 52242

JACINTO, ESTELA, PHD
PROFESSOR
DEPARTMENT OF BIOCHEMISTRY & MOLECULAR BIOLOGY
ROBERT WOOD JOHNSON MEDICAL SCHOOL
RUTGERS UNIVERSITY
PISCATAWAY, NJ 08854

JIANG, YU, PHD
PROFESSOR
DEPARTMENT OF PHARMACOLOGY & CHEMCIAL BIOLOGY
UNIVERSITY OF PITTSBURGH SCHOOL OF MEDICINE
PITTSBURGH, PA 15261

KARELLAS, ANDREW, PHD
PROFESSOR AND DIRECTOR
BIOMEDICAL IMAGING INNOVATION
DEPARTMENT OF MEDICAL IMAGING
UNIVERSITY OF ARIZONA
TUCSON, AZ 85724

KEATING, NANCY L, MD
PROFESSOR
DEPARTMENT OF HEALTH CARE POLICY
HARVARD MEDICAL SCHOOL
BOSTON, MA 02115

KOYA, RICHARD C, MD, PHD
PROFESSOR
UNIVERSITY OF CHICAGO SCHOOL OF MEDICINE
DEPARTMENT OF OBSTRETICS AND GYNECOLOGY
IRECTOR OF THE CGMP VECTOR DEVELOPMENT PRODUCTION FACILITY
CHICAGO, IL 14263-6063

LUSTBERG, MARYAM B, MD
ASSOCIATE PROFESSOR
INTERNAL MEDICINE-MEDICAL ONCOLOGY
MEDICAL DIRECTOR OF SUPPORTIVE CARE
THE OHIO STATE UNIVERSITY COMPREHENSIVE CANCER CTR
COLUMBUS, OH 43210

MUSCAT, JOSHUA E, PHD
PROFESSOR
DEPARTMENT OF PUBLIC HEALTH SCIENCES
PENN STATE COLLEGE OF MEDICINE
MILTON S HERSHEY MEDICAL CENTER
HERSHEY, PA 17033

NAKSHATRI, HARIKRISHNA, BVSC, PHD
PROFESSOR
DEPARTMENT OF SURGERY
INDIANA UNIVERSITY SCHOOL OF MEDICINE
INDIANAPOLIS, IN 46202

PAYTON, JACQUELINE E, MD, PHD
ASSISTANT PROFESSOR
DEPARTMENT OF PATHOLOGY AND IMMUNOLOGY
CO-DIRECTOR, PATHOLOGY PHYSICIAN SCIENTIST PROG
WASHINGTON UNIVERSITY SCHOOL OF MEDICINE
SAINT LOUIS, MO 63110

RIEHN, ROBERT, PHD
PROFESSOR OF PHYSICS
DEPARTMENT OF PHYSICS
NORTH CAROLINA STATE UNIVERSITY
RALEIGH, NC 27695

RUTTEN, LILA J, PHD
PROFESSOR OF HEALTH SERVICES RESEARCH
DIVISION OF HEALTH CARE DELIVERY RESEARCH
KERN CENTER- SCIENCE OF HEATLH CARE DELIVERY
MAYO CLINIC
ROCHESTER, MN 55905

SCARPINATO, KARIN D, PHD
SENIOR ASSOCIATE VICE PRESIDENT
DIRECTOR, POSTDOCTORIAL AFFAIRS
DIVISON OF RESEARCH
FLORIDA ATLANTIC UNIVERSITY
BACO RATON, FL 33431

SIRACUSA, LINDA D, PHD
PROFESSOR
DEPARTMENT OF MEDICAL SCIENCES
HACKENSACK MERIDIAN SCHOOL OF MEDICINE
NUTLEY, NJ 07110

TSUI, JENNIFER, PHD
DEPARTMENT OF PREVENTIVE MEDICINE
KECK SCHOOL OF MEDICINE
UNIVERSITY OF SOUTHERN CALIFORNIA
LOS ANGELES, CA 90089

ZHANG, RUIWEN, MD, PHD
PROFESSOR OF PHARMOCOLOGY AND TOXICOLOGY
DIRECTOR OF UH DRUG DISCOVERY INSTITUTE
UNIVERSITY OF HOUSTON
HOUSTON, TX 77204

SCIENTIFIC REVIEW OFFICER

CHEN, SCOTT A., PHD
SCIENTIFIC REVIEW OFFICER
DIVISION OF EXTRAMURAL ACTIVITIES
NATIONAL CANCER INSTITUTE
NATIONAL INSTITUTES OF HEALTH
ROCKVILLE, MD 20850

EXTRAMURAL SUPPORT ASSISTANT

CRAIGIE, JANET L
PROGRAM ANALYST
DIVISION OF EXTRAMURAL ACTIVITIES
NATIONAL CANCER INSTITUTE
NATIONAL INSTITUTES OF HEALTH
ROCKVILLE, MD 20850

PROGRAM REPRESENTATIVE

ELJANNE, MARIAM
PROGRAM REPRESENTATIVE
DIVISION OF CANCER BIOLOGY
OFFICE OF THE DIRECTOR
NATIONAL CANCER INSTITUTE
ROCKVILLE, MD 20892-9747

Consultants are required to absent themselves from the room during the review of any application if their presence would constitute or appear to constitute a conflict of interest.
